# The effectiveness of an Australian community suicide prevention networks program in preventing suicide: a controlled longitudinal study

**DOI:** 10.1186/s12889-022-14331-1

**Published:** 2022-10-19

**Authors:** A. J. Morgan, R. Roberts, A. J. Mackinnon, L. Reifels

**Affiliations:** 1 Centre for Mental Health, Melbourne School of Population and Global Health, University of Melbourne, 3010 Carlton, VIC Australia; 2 Centre for Urban Research, School of Global, Urban and Social Studies, RMIT University, 3000 Melbourne, VIC Australia

**Keywords:** Suicide, Suicide prevention, Community networks

## Abstract

**Background:**

Suicide is a major issue affecting communities around the world. Community-based suicide prevention approaches can tailor activities at a local level and are recognised as a key component of national suicide prevention strategies. Despite this, research exploring their effects on completed suicides is rare. This study examined the effect of a national program of community suicide prevention networks on suicide rates in catchment areas across Australia.

**Methods:**

Australian suicide data from the National Coronial Information System for 2001–2017 were mapped to geographic catchment areas of community suicide prevention networks and matched control areas with similar characteristics. The effect of network establishment on suicide rates was evaluated using longitudinal models including fixed effects for site type (network or control), time, season, and intervention (network establishment), with site included as a random intercept.

**Results:**

Sixty suicide prevention networks were included, servicing areas with a population of 3.5 million. Networks varied in when they were established, ranging from 2007 to 2016. Across the time-period, suicide rates per 100,000 per quarter averaged 3.73 (SD = 5.35). A significant reduction in the suicide rate of 7.0% was found after establishment of networks (IRR = 0.93, 95% CI 0.87 to 0.99, p = .025).

**Conclusion:**

This study found evidence of an average reduction in suicide rates following the establishment of suicide prevention networks in Australian communities. These findings support the effectiveness of empowering local communities to take action to prevent suicide.

## Background

Suicide is recognised as a public health crisis, both in Australia [1] and around the world [2]. Suicide has multiple causes, and effective suicide prevention requires a multifaceted strategy. Community-based approaches are an important component of national strategies in suicide prevention, as they can take an integrated and coordinated approach at a local level [3]. Community-based approaches vary from smaller-scale community-education interventions that focus on reducing stigma and increasing help-seeking [4], through to multi-level interventions, such as the Alliance Against Depression [5], which includes training of primary care providers, public awareness campaigns, gatekeeper training, and interventions for at-risk individuals. While early evaluations of multi-level interventions showed promise as an effective means of suicide prevention [6], more recent research is equivocal [5, 7].

Strong evidence for other types of community-based approaches for suicide prevention is limited, with most research focusing on knowledge and attitudinal outcomes or proxy outcomes such as suicidal ideation [4, 8]. An exception is the Garrett Lee Smith youth suicide prevention program, a community-level intervention with evidence supporting its effectiveness [9, 10]. This is a United States government-funded program that targets suicide reduction in young people. Counties that receive funding implement a range of local suicide prevention activities, with an emphasis on gatekeeper training. Analyses have shown a reduction in youth suicides up to 2 years after the end of program implementation, with effects fading after 3 years [9]. These findings support the importance of tackling suicide within local communities and highlight the need for sustainable delivery of suicide prevention initiatives to maintain effects.

Within Australia, there is a renewed focus on community-based approaches to suicide prevention [11]. Despite suicide prevention being a priority in Australian mental health policy, suicide rates are not decreasing, and in fact, have increased over the past decade [12]. There have also been growing calls for a systems-based approach to suicide prevention that includes multi-level interventions implemented simultaneously in local communities [13]. In light of this, the Australian Government funded the implementation and evaluation of a multi-level systems approach to suicide prevention in 12 regions across Australia as part of the National Suicide Prevention Trial [14]. These were coordinated by the government-funded regional Primary Health Network, which provides general practitioner and community based allied health services. Although a range of positive outcomes were reported, initial findings do not provide empirical support for a reduction in suicides during the trial period [14].

The Wesley LifeForce Networks program is another model of community-based suicide prevention. The program is an initiative of Wesley Mission, a major nongovernmental organisation that provides secular community support services in Australia. It aims to empower local communities to take action to prevent suicide by working collaboratively with community members to develop a sustainable local suicide prevention network [15]. During each local program’s establishment phase, the national organisation supports the local network to bring together stakeholders that have an interest or mandate in suicide prevention, assists in identifying key issues in the community and helps develop a strategic plan and activities to prevent suicide at a local level. Networks aim to fill a community-identified support gap in areas of higher need and avoid duplication of programs. Network activities are therefore tailored to local contexts, but there is a shared focus on interagency cooperation and raising community awareness. Common activities across networks include distributing support service information, facilitating community access to support services, and organising suicide prevention training and community awareness and anti-stigma initiatives [16]. These upstream capacity building initiatives complement other suicide-prevention activities led by service providers such as primary health. While each Wesley LifeForce Network is community-led, they also receive ongoing assistance from a national team of community development coordinators, including advice, information, administrative and operational and governance support, and can apply for small amounts of seed funding [17]. There are over 100 Networks across Australia, particularly in high-risk communities where there is a greater need (e.g., regional or remote communities, [18]). Although the program has degrees of similarity with other suicide prevention initiatives, there are four aspects that together set it apart: Networks are community-led (not just community-based); networks do not impose on communities a pre-existing model of suicide prevention interventions; the program operates as a network of Networks with national support; and there is a very large number of participating Networks. The Wesley LifeForce program is therefore unique in Australia and worldwide, as the only nationally operating non-government program supporting suicide prevention networks at a grassroots level [19].

Although community-based approaches to suicide prevention are often recommended [3], research evaluating their impact on suicide rates is rare. To the best of our knowledge, no previous studies have examined the ultimate outcomes of a similar model of community-led suicide prevention networks in terms of a reduction in suicides. Other community-based suicide prevention initiatives that have been evaluated are typically structured interventions (which can be partly tailored to local contexts) or are delivered in a single community [20]. The size of the Wesley LifeForce Networks program presents a unique opportunity to explore the effect of community-led suicide prevention on completed suicides, rather than proxy suicide outcomes. This study therefore aimed to examine the effect of the establishment of Wesley LifeForce Networks across Australia on the suicide rate in Network catchment areas. We hypothesized that suicide rates would show a decrease following Network establishment across the national cohort of Networks, controlling for suicide rates in matching control communities without a Wesley LifeForce Networks program.

## Methods

### Sampling of wesley lifeforce networks

As of 2019, there were 92 Networks in operation, with about a third of these established in 2017 or later [16]. To be included, LifeForce Networks had to be operational and established between 2001 and before 2017, leaving 60 Wesley LifeForce Networks included. There were more Networks in regional areas (n = 30, 50%) than in major cities (n = 18, 30%) or remote areas (n = 12, 20%), which is consistent with the profile of all Networks [19]. The distribution of Networks across Australian state or territories was also broadly representative of the profile of all Networks. The Networks serviced areas with a total population of 3,500,951 (averaged across the time period), with a median population of 28,884. The first Network was established in 2007 and the most recent one in 2016, with 30 (50%) established in 2014 or later.

### Data collection

Suicide counts were obtained from the National Coronial Information System (NCIS) on all closed cases of intentional self-harm (with a final determination of suicide) that had been notified to a coroner between 2001 and 2017. The NCIS is an online data repository for all external cause deaths in Australia. The completeness of the NCIS data (i.e. cause of death has been determined and the coroner has made a finding) ranged from 95.8 to 99.0% across the study period. Data after 2017 were not included because it can take up to 3 years for a case to be closed. Date of notification and residential location were collected on each case. The geographic location and catchment area size of LifeForce Networks were provided by Wesley Mission and confirmed with each Network wherever possible. General demographic data of LifeForce Network catchment areas and control communities were obtained from the Australian Bureau of Statistics (ABS) on the population size, remoteness category (major city, inner regional, outer regional, remote and very remote, Australian Bureau of Statistics [21]), and relative socio-economic disadvantage, which is an index that ranks areas based on household income, qualification and occupation [22].

The study was approved by the University of Melbourne Human Research Ethics Committee (Ethics ID 1954813.3). Ethics approval was also obtained from the NCIS Research Committee (MO446), the Victorian Department of Justice and Community Safety Human Research Ethics Committee (CF/20/6638), the Coroners Court of Victoria Research Committee (RC 344), and the Western Australian Coronial Ethics Committee (EC02/2019).

### Data mapping and selection of control areas

Each LifeForce Network provided us with their postcode and a Geographic Information System (GIS) and 2016 ABS suburb and postcode digital boundaries were used to model the catchment areas of Networks. As some postcodes cover large areas and contain several suburbs, catchment areas for each Network were modelled by selecting all suburbs which intersected the postcode area. Each LifeForce Network was provided with a list of suburb names which provisionally represented their catchment area and asked to review the data by confirming, deleting, or adding additional suburbs where necessary. The catchment areas of LifeForce Networks which provided feedback were amended in the GIS as appropriate and are denoted as ‘boundary confident’ in the following. Suicide data compiled from the NCIS were matched to Networks and control areas based on the ABS Statistical Areas Level 2 (SA2)^1^ code of the person’s residence.

Control areas without established LifeForce Networks but with similar demographic characteristics were identified and matched to LifeForce Networks at a ratio of 1:1, based on key criteria including, remoteness, relative socio-economic disadvantage, and population size, using ABS demographic data from the 2016 Census [22]. To maintain similar catchment area sizes across LifeForce Networks and control areas, ABS Statistical Areas Level 3 (SA3)^2^ were used to model control areas in metropolitan areas and ABS SA2 statistical areas were used to model control areas in regional and remote areas.

The characteristics of the 60 control areas were similar to Network areas. There was no significant difference in socio-economic disadvantage scores between Network and control areas, t(59)=-1.74, p = .087. Networks and controls also matched perfectly for remoteness area. Mean population was significantly lower in control areas (M = 29,724, SD = 39,359) than Network areas (M = 58,349, SD = 71,386), t(59) = 3.52, p < .001.

### Data analysis

Mixed effects longitudinal models for count data models were developed to examine the effect of Network establishment on suicide rates. Due to the frequency of months with zero suicides, data were aggregated to form a quarterly count of suicides per site. The effect of individual sites was included as a random intercept. Suicide counts were modelled as a Poisson distribution as a preliminary likelihood-ratio test showed that the alternative negative binomial distribution was not significantly superior. The model contained fixed effects for site type (Network or control), intervention status, and time. Intervention status referred to whether and for how long a network had been in operation for a particular site and was implemented as one or more binary indicator variables to model change at different time after establishment of a Network at a site.

Given evidence of a non-linear suicide rate over 2001–2017, the form of the time trend was chosen using fractional polynomials implemented with the fp command in Stata. This allows for a wide variety of functional forms [23] and showed that a trend including linear and quadratic terms was the best fitting. This indicated a decrease in the suicide rate followed by an increase, which is consistent with national data for the time period [12]. As suicide rates show a seasonal pattern, with higher rates in Spring (September to November in Australia) or early Summer [24, 25], models included a variable for quarter in the year, to adjust for differential patterns of suicide rates across the calendar year. Site population size was included as an exposure variable so that model parameters of suicide counts could be interpreted as rates. Population size was calculated from the Australian Bureau of Statistics, which provides annual population data disaggregated by SA2 or SA3 [26].

We first investigated a model testing whether introducing LifeForce Networks led to a step change in suicide rates, whereby the intervention effect was modelled as an indicator variable with a value of 0 up to establishment and 1 thereafter. We then explored the pattern of non-constant change in suicide rates attributable to introducing the program. While the gradually increasing effect of an intervention is a plausible mode of change, an unconstrained effect is implausible: it would imply that suicide rates continue to decrease every quarter after network establishment. Accordingly, time after network establishment was dummy coded as either quarters 1 to 4 (covering the first year after establishment) or those quarters beyond the first year. Quarters before network establishment were the reference category. For control areas, all quarters were coded as zero (reference). We examined whether any site was particularly influential on model parameters by using a jackknife approach to estimate the model leaving out one site at a time.

Effects are expressed as incidence rate ratios (IRR). IRRs less than 1 indicate a decrease in suicide rates and IRRs greater than 1 indicate an increase. The significance level was set at p < .05. Analyses were conducted in Stata 16.0 (College Station, TX: StataCorp LLC).

## Results

Each site (Network or control) contributed 68 observations and there was data available on suicide rates for at least 4 quarters after Network establishment for all Networks.

Across Network sites, the number of suicides over the period totalled 7,903. Suicide rates per 100,000 per quarter averaged 3.73 (SD = 5.35) and ranged between 0 and 65.06. There was substantial variation in suicide rates between Networks, with mean rates from 1.55 to 12.79. Across control sites, there were 3,446 suicides over the period. Suicide rates per 100,000 per quarter were somewhat lower than in Network areas (M = 3.53, SD = 7.70), consistent with Networks being established in communities with greater need.

**Table 1:** Step change in suicide rates after Network establishment

	IRR	95% CI	p
Time (linear)^a^	0.9925	0.9877 to 0.9972	0.002
Time (quadratic)^b^	1.0001	1.0000 to 1.0002	0.001
Site type (Network vs. control)	1.14	1.00 to 1.30	0.051
Season (Quarter in year^c^)			
1st	0.99	0.94 to 1.04	0.645
2nd	0.91	0.87 to 0.96	< 0.001
3rd	0.96	0.91 to 1.01	0.090
Network establishment	0.93	0.87 to 0.99	0.025

a. Measured in elapsed quarters since January 2001, power is 1

b. Measured in elapsed quarters since January 2001, power is 2

c. Quarter 4 is the reference category

Table 1 presents the estimates from the step change model. The background temporal trend showed an initial linear decrease and then a very small quadratic increase in the suicide rate over time. There was also evidence of a seasonal effect, with lower suicide rates in the second quarter of the year compared to the fourth quarter. The fixed effect of site was close to statistically significant, consistent with the rationale for establishing Networks in areas of greater need. On average, the introduction of Wesley LifeForce Networks reduced the suicide rate by 7%, indicated by an IRR of 0.93. Suicide rates before networks were introduced averaged 3.74 per 100,000 per quarter, and afterwards averaged 3.48 per 100,000 per quarter. This equates to 1.04 fewer deaths per 100,000 per year. Furthermore, there were no individual sites that had a large influence on the effect of network establishment. A supplementary analysis that restricted sites to those where the boundary was confirmed (37 Network sites plus matching control sites) also showed consistent findings (Network establishment IRR = 0.91, 95% CI 0.85 to 0.96, p = .002).

The data were further explored to investigate incremental change after Networks were established in each community. The pattern of effects suggested that reductions in suicides peaked in the third quarter after Network establishment, where there was a significant reduction of 17% in suicide rates (see Table 2).

**Table 2:** Incremental change in suicide rates after Network establishment

	IRR	95% CI	p
Time (linear)^a^	0.9925	0.9876 to 0.9975	0.003
Time (quadratic)^b^	1.0001	1.0000 to 1.0002	0.001
Site (Network vs. control)	1.14	1.00 to 1.30	0.052
Season (Quarter in year^d^)			
1st	0.99	0.94 to 1.04	0.640
2nd	0.91	0.87 to 0.96	0.001
3rd	0.96	0.91 to 1.01	0.092
Time after Network establishment^c^			
1st quarter	0.96	0.78 to 1.18	0.677
2nd quarter	0.96	0.78 to 1.18	0.701
3rd quarter	0.83	0.71 to 0.98	0.030
4th quarter	0.90	0.75 to 1.09	0.281
5th quarter onwards	0.94	0.87 to 1.01	0.091

a. Measured in elapsed quarters since January 2001, power is 1

b. Measured in elapsed quarters since January 2001, power is 2

c. Before Network establishment is the reference category

d. Quarter 4 is the reference category

## Discussion

This study has demonstrated that a nationally-supported program of community-led suicide prevention networks was associated with fewer suicides in network communities. A reduction in the suicide rate was observed following the establishment of Networks across a national cohort of 60 Wesley LifeForce Networks. The effect was somewhat smaller than found in other community-based suicide prevention initiatives, such as the Garrett Lee Smith program, which showed 1.33 fewer deaths per 100,000 in the year following the program [10], as compared with 1.04 in this study. Although the size of the effect is relatively small, given the deleterious impact of suicide on social networks and communities [27, 28] and the significant scale of the Wesley LifeForce program, this effect may have important public health impact. It also approaches the World Health Organization goal of reducing the suicide rate by 10% [2].

The pattern of change in suicide rates after Networks were established was also examined to explore whether there were incremental non-linear effects, such as an initial reduction followed by maintenance of effects or a deterioration in effects. Results tentatively suggested that effects were strongest 6–9 months following the establishment of a Network, followed by some reduction in impact. The reason for this particular pattern is unclear. It is possible that this is the result of a burst of Network activity in the first 6 months of establishment, when motivation and initial momentum was high, but other data on Network activities would be required to support this mechanism. Unlike other suicide prevention interventions, which have a fixed duration of program implementation and show a deterioration of effects after the intervention ends [9], Wesley LifeForce Networks are designed to be sustainable, ongoing initiatives. It is therefore important to understand how Networks can sustain the initial momentum and commitment of members over many years for maximum impact. Efforts to improve ongoing data collection of Network processes and activities may assist in identifying key mechanisms of impact. A survey of Network coordinators has suggested that several internal Network processes may be important predictors of outcomes, including holding more frequent meetings and regularly identifying relevant community stakeholders [16]. Networks that had existed for longer were also associated with better perceived outcomes. These factors are consistent with the broader literature on community coalition effectiveness in health promotion [29].

The Wesley LifeForce Networks program model provides a vehicle to bring together local stakeholders to advance suicide prevention via locally targeted initiatives. The Wesley ‘network of networks’ model provides economies of scale in operational support and governance structures, while providing individual networks the flexibility to address locally relevant risk factors for suicidality. Our findings suggest that this model is an effective means to broaden community engagement and foster a whole-of-community approach to suicide prevention, via up-stream initiatives focused on raising community awareness, reducing stigma, supporting others, fostering connections, providing information, training or capacity building. Although this flexibility may be a key strength of the program, the absence of a uniformly structured intervention program does increase the challenge of identifying the mechanisms or most effective suicide prevention activities. As Networks are largely run by volunteers with limited resources, a greater understanding of what is likely to work and under what conditions would help Networks decide which activities should be prioritised within their communities.

This study had a number of strengths, including the analysis of 17 years of suicide data across multiple community suicide prevention networks. While Network establishment was not randomized, the inclusion of control communities aimed to reflect the contemporaneous trajectory of suicides in the absence of intervention. Nevertheless, our findings should be considered in light of study limitations. While Network and control areas were well matched on several key criteria, the analysis did not account for other factors, such as existing health service arrangements or any other specific suicide prevention programs. Not all Networks could be included in the analysis, as there was a lack of post-establishment data on suicide for more recently established Networks. Nevertheless, the sample of Networks was representative of the population of Networks in terms of remoteness and State. Furthermore, while catchment area boundaries could not be confirmed for all Networks, the analysis was generally based on conservative Network boundary estimates. Sensitivity analyses conducted with the subsample of Networks where we had confirmation of geographic boundaries also supported overall findings.

## Conclusion

In conclusion, study findings suggest that supporting and empowering local communities to take action to tackle suicide can help prevent suicide. These findings may be useful to other community-based suicide prevention initiatives and can inform suicide prevention policy.

## Data Availability

Data from this study is not available for sharing. While data custodian policies do not permit public sharing of study data, interested parties can apply for access to national suicide data from the NCIS at www.ncis.org.au.

## Footnotes

1 Statistical Areas Level 2 (SA2s) are medium-sized geographic areas with a population generally between 3,000 and 25,000 that represent a community that interacts together socially and economically.

2 Statistical Areas Level 3 (SA3s) are geographic areas built from Statistical Areas Level 2 (SA2s) and generally have populations between 30,000 and 130,000 people.
